# Dataset of trend-preserving bias-corrected daily temperature, precipitation and wind from NEX-GDDP and CMIP5 over the Qinghai-Tibet Plateau

**DOI:** 10.1016/j.dib.2020.105733

**Published:** 2020-05-21

**Authors:** Shuo Chen, Weihang Liu, Tao Ye

**Affiliations:** 1 Key Laboratory of Environmental Change and Natural Disaster, Ministry of Education, Faculty of Geographical Science, Beijing Normal University, Beijing, 100875, China; 2 School of Geographical Sciences, Guangzhou University, Guangzhou, 510006, China; 3 State Key Laboratory of Earth Surface Processes and Resource Ecology, Ministry of Education, Faculty of Geographical Science, Beijing Normal University, Beijing, 100875, China; 4 Academy of Disaster Reduction and Emergency Management, Ministry of Emergency Management & Ministry of Education, Beijing, 100875, China

**Keywords:** NEX-GDDP, CMIP5, bias correction, Qinghai-Tibet Plateau

## Abstract

A bias-corrected dataset containing daily meteorological data over the Qinghai-Tibet Plateau has been generated using a trend-preserving bias correction, the Inter-Sectoral Impact Model Intercomparison Project (ISI-MIP) approach, with a high-quality gridded meteorological dataset based on ground observations (CN05.1). The dataset contains daily bias-corrected values of maximum/minimum near-surface air temperature, precipitation and mean near-surface wind speed from 15 models from the Fifth Phase of the Coupled Model Intercomparison Project (CMIP5) and a downscaled high-resolution dataset (NEX-GDDP), based on CMIP5 models, over the Qinghai-Tibet Plateau (QTP) during 1986–2095. This dataset provides an important reference for the study of future climate change and its impacts in the Qinghai-Tibet Plateau region.

Specifications table

**Table:** 

Subject	Global and Planetary Change; Geology
Specific subject area	Climate change; Natural disasters
Type of data	NetCDF
How data were acquired	NEX-GDDP/CMIP5 data were downloaded from their websites (links are provided in the “Description of data collection” section). A trend-preserving bias correction was applied to calibrate them. They were further processed to produce annual and seasonal mean and extreme values, to assess the skill of the bias correction. Matlab2019b and ArcGIS10.3 were the main tools used for data processing.
Data format	Raw and analyzed
Parameters for data collection	NEX-GDDP: 0.25° × 0.25° spatial resolution, training period 1986–2005, future period 2006–2095/2099/2100 (varies by model), daily temporal resolution, raster data. CMIP5: spatial resolution varies by model (see Table 1), training period 1986–2005, future period 2006–2100, daily temporal resolution, raster data. CN05.1: 0.25° × 0.25° spatial resolution, training period 1986–2005, daily temporal resolution, raster data.
Description of data collection	Maximum near-surface air temperature (Tmax), minimum near-surface air temperature (Tmin) and precipitation (Pr) were downloaded from the NEX-GDDP website (url: https://nex.nasa.gov/nex/projects/1356/) and mean near-surface wind speed (Wind) was downloaded from the CMIP5 website (url: https://esgf-node.llnl.gov/search/cmip5/). CN05.1 data were obtained from the China Meteorology Administration's National Climate Center. More details can be found in Refs. [2], [3], [4].
Data source location	The main body of the Qinghai-Tibet plateau in the range of 26°N–39.75°N, 73.25°E–104.75°E.
Data accessibility	Raw data are deposited in the Zenodo repository: Tmax Data identification number: 10.5281/zenodo.3747017 Direct URL to data: https://doi.org/10.5281/zenodo.3747017 Tmin Data identification number: 10.5281/zenodo.3748094 Direct URL to data: https://doi.org/10.5281/zenodo.3748094 Pr Data identification number: 10.5281/zenodo.3745947 Direct URL to data: https://doi.org/10.5281/zenodo.3745947 Wind Data identification number: 10.5281/zenodo.3748954 Direct URL to data: https://doi.org/10.5281/zenodo.3748954 Analyzed data are available in this article as supplementary files.

## Value of the data

• This dataset includes daily maximum/minimum near-surface air temperature, precipitation and mean near-surface wind speed from 15 models under the RCP4.5 and RCP8.5 scenarios. Its accuracy is further improved by being bias-corrected using local observation data (CN05.1), containing more observation gauges.
• This dataset is of great value to researchers who study the impacts and risks of climate change in the QTP. Its spatial and temporal resolution allows potential users to develop their impact or risk assessment models at a higher resolution in this region.
• For further insights and development of experiments, for example, it can be used to predict future extreme weather events, crop yields, etc. Furthermore, using data from 15 models enables multi-model ensemble analysis, which is considered to be essential in climate projection and climate risk analysis.

## 1 Data Description

In the following, “NEX-GDDP/GCMs before bias correction” refers to the original data that we downloaded from the relevant websites and “NEX-GDDP/GCMs after bias correction” refers to the data that we have bias-corrected by applying the ISI-MIP approach.

The raw dataset contains daily maximum/minimum near-surface air temperature, precipitation and mean near-surface wind speed from 15 models (see Table 1), which were bias-corrected using the ISI-MIP approach [1] under two RCP scenarios (RCP4.5 and RCP8.5). The resolution is 0.25° × 0.25°. The data are stored in NetCDF format. The download links are provided in the section “Data accessibility”.

**Table 1:** The 15 selected climate models

Model	Institution	Country	Number of grid points	
			CMIP5	NEX-GDDP
ACCESS1-0	CSIRO-BOM	Australia	192 × 145	1440 × 720
CanESM2	CCCma	Canada	128 × 64	1440 × 720
CNRM-CM5	CNRM-CERFACS	France	256 × 128	1440 × 720
CSIRO-Mk3-6-0	CSIRO-QCCCE	Australia	192 × 96	1440 × 720
GFDL-CM3	NOAA-GFDL	USA	144 × 90	1440 × 720
GFDL-ESM2G	NOAA-GFDL	USA	144 × 90	1440 × 720
GFDL-ESM2M	NOAA-GFDL	USA	144 × 90	1440 × 720
INM-CM4	INM	Russia	180 × 120	1440 × 720
IPSL-CM5A-LR	IPSL	France	96 × 96	1440 × 720
IPSL-CM5A-MR	IPSL	France	144 × 143	1440 × 720
MIROC5	MIROC	Japan	256 × 128	1440 × 720
MIROC-ESM	MIROC	Japan	128 × 64	1440 × 720
MPI-ESM-LR	MPI-M	Germany	192 × 96	1440 × 720
MPI-ESM-MR	MPI-M	Germany	192 × 96	1440 × 720
MRI-CGCM3	MRI	Japan	320 × 160	1440 × 720

For analyzed data, we processed the daily bias-corrected data into annual, winter (from December to next February, DJF) and summer (from July to August, JJA) values and then calculated the differences between NEX-GDDP/GCM data before/after bias correction and CN05.1 (i.e., multi-model minus CN05.1) to show the improvement from applying the bias correction process. For mean values, we use annual, winter and summer average of Tmax, Tmin, Pr, and Wind. For climate extreme values, we use the 95th percentile of Tmax, the 5th percentile of Tmin, the 95th percentile of Pr and the 95th percentile of Wind. The trend values are represented by the slope of the unary linear regression of the observed/multi-model time series.

The rest of this section shows the differences between the NEX-GDDP/GCM data before/after bias-correction and the reference data.

### 1.1 Differences between NEX-GDDP/GCM data and observations (CN05.1) before and after bias correction during the training period (1986–2005)

**Fig. 1:**
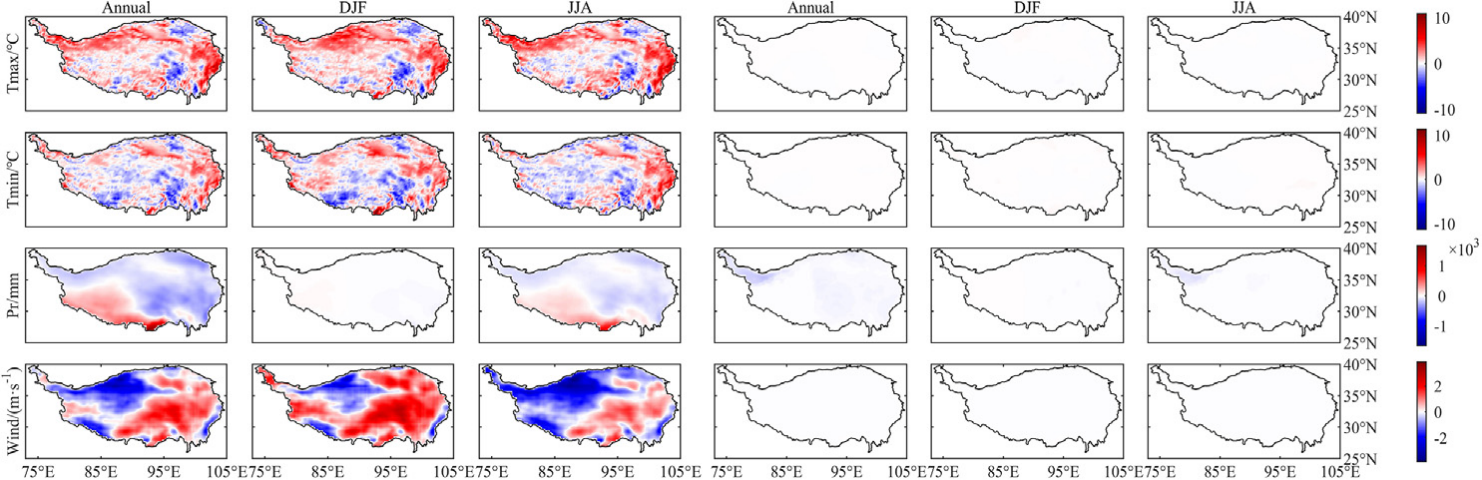
Differences (NEX-GDDP/GCMs minus CN05.1) in mean values during the training period (1986–2005). The left three columns show values before bias correction and the right three columns show values after bias correction. Units: Tmax (°C), Tmin (°C), Pr (mm), Wind (m·s^−1^). Data shown here are from averages over 15 models.

**Fig. 2:**
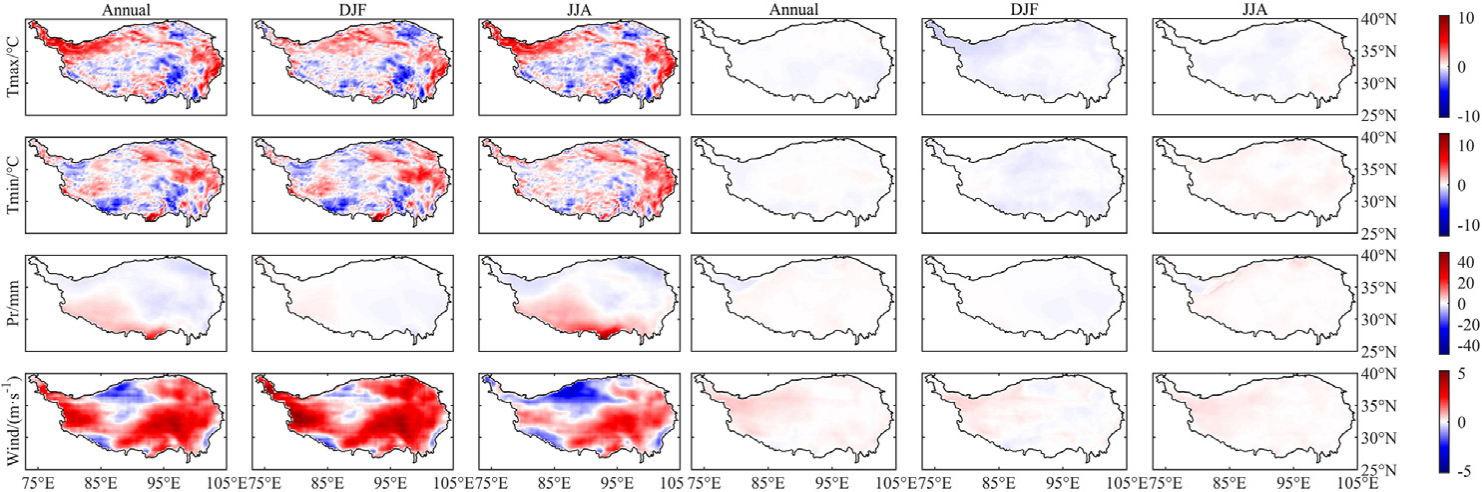
Differences (NEX-GDDP/GCMs minus CN05.1) in extreme values during the training period (1986–2005). The left three columns show values before bias correction and the right three columns show values after bias correction; Units: Tmax (°C), Tmin (°C), Pr (mm), Wind (m·s^−1^). Data shown here are from averages over 15 models.

As can be seen from Figs. 1 and 2, there are large spatial differences between the models and CN05.1 before the bias correction is applied. Although the NEX-GDDP data have already been bias-corrected using GMFD (Global Meteorological Forcing Dataset) during their generation process, it is still necessary to correct the biases in this dataset using CN05.1, which contains more observation gauges over the study area. After bias correction, the agreement between the dataset and CN05.1 for the training period has substantially improved.

### 1.2 Changes during the future period (2006–2095) under RCP4.5, after bias correction

**Fig. 3:**
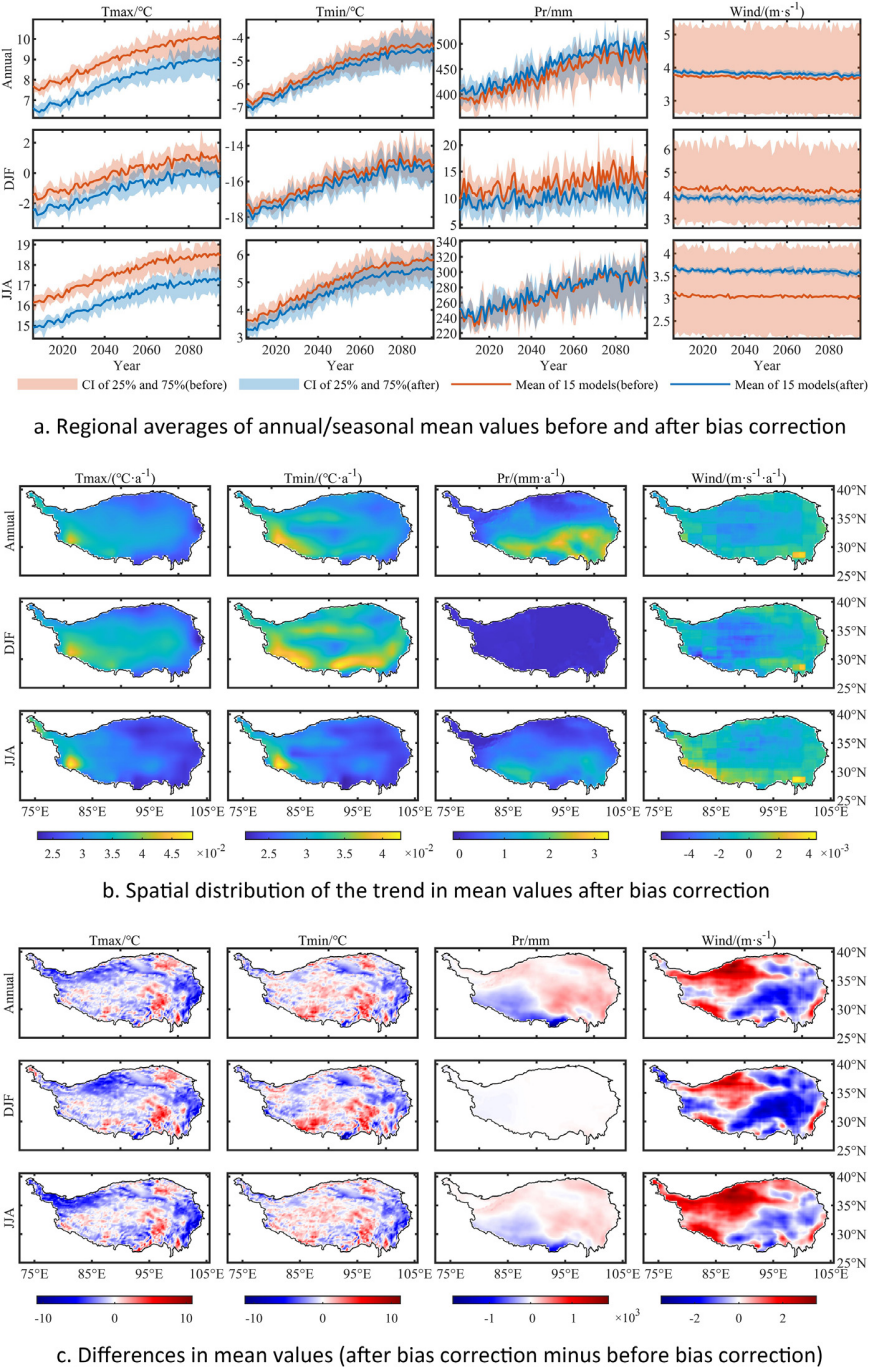
Mean values from NEX-GDDP/GCM data from 2006 to 2095 under RCP4.5, before and after bias correction. Units: Tmax (°C), Tmin (°C), Pr (mm), Wind (m·s^−1^); Data shown here are from averages over 15 models.

**Fig. 4:**
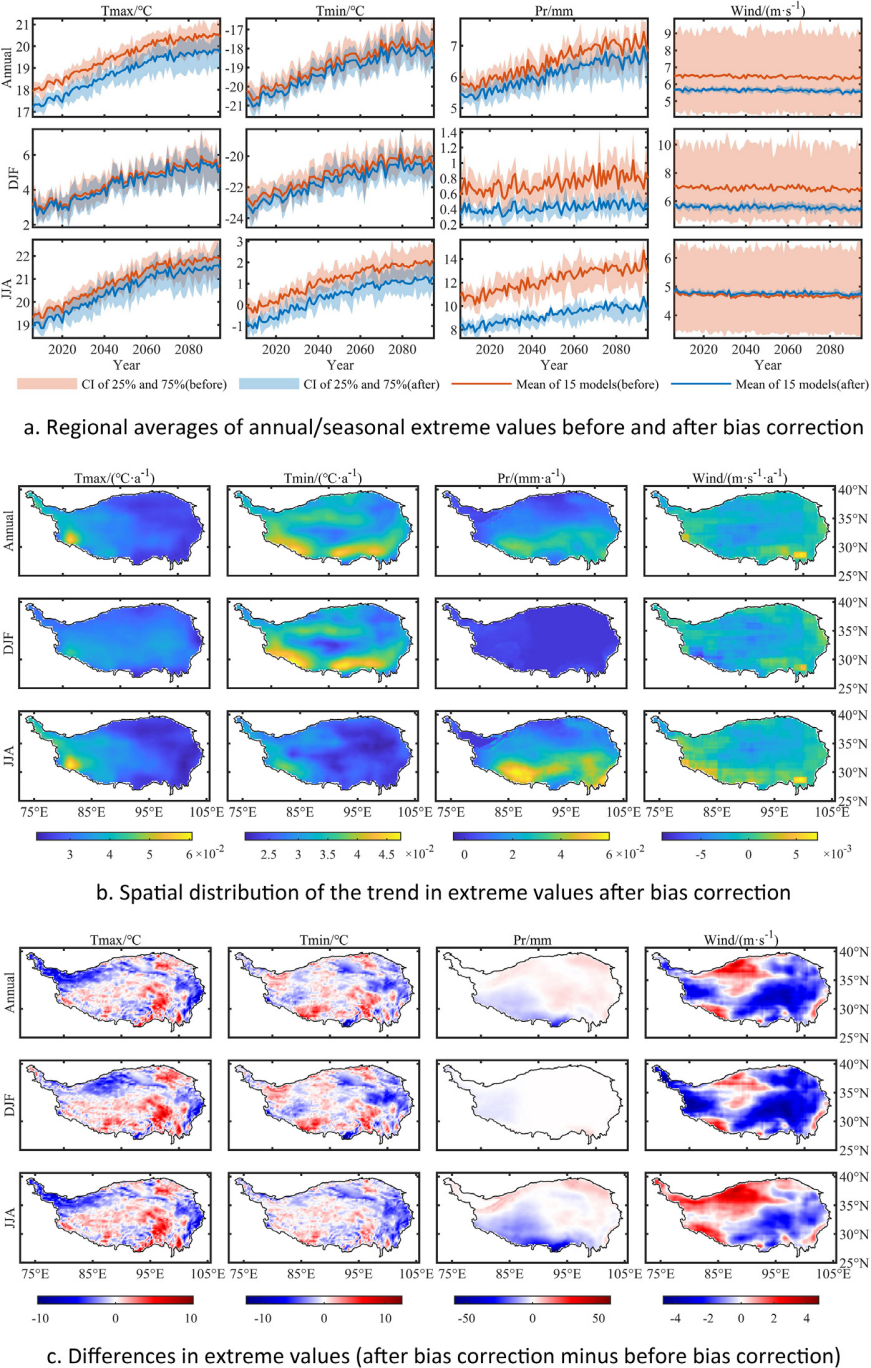
Extreme values from NEX-GDDP/GCM data from 2006 to 2095 under RCP4.5, before and after bias correction. Units: Tmax (°C), Tmin (°C), Pr (mm), Wind (m·s^−1^); Data shown here are from averages over 15 models.

For the future period, the bias correction process adjusts the values to better relate to historical values (Figs. 3a and 4a), while the long-term trend, which is represented by the linear regression slope, is well preserved (Figs. 3b and 4b). The spatial variation of the differences between the models before and after bias correction are shown in Figs. 3c and 4c.

## 2 Experimental Design, Materials and Methods

The models to be bias-corrected contain two sets of simulated data. Fifteen models were selected, with data from a training period (1986–2005), a validation period (1966–1985) and a future period (2006–2095/2099/2100) under two Representative Concentration Pathway (RCP) scenarios, 4.5 and 8.5. Tmax, Tmin and Pr were obtained from NEX-GDDP and Wind was obtained from CMIP5. Tmax and Tmin are at a height of 2 meters and Wind is at a height of 10 meters. Near-surface wind is important to climate change in terms of the carbon exchange, water transport, evapotranspiration, etc., but NEX-GDDP does not contain this variable. We aim to provide a dataset containing interpolated and bias-corrected wind speeds that are convenient to use in research. NEX-GDDP is a statistical downscaling dataset based on CMIP5. In our dataset, Tmax, Tmin and Pr (from NEX-GDDP) and Wind (from CMIP5) come from the same 15 models and the same ensemble (r1i1p1).

The reference dataset is CN05.1 [4], a daily gridded dataset released by the China Meteorology Administration's National Climate Center, including Tmax, Tmin, Pr and Wind. Tmax and Tmin are at a height of 2 meters and Wind is at a height of 10 meters. The spatial resolution is 0.25° × 0.25° and the data cover the period from 1st January 1966 to 31st December 2005.

The region studied extends from 73.25°E to 104.75°E and from 26°N to 39.75°N. CMIP5 data were interpolated into a 0.25° × 0.25° grid using a nearest-neighbor interpolation method. We divided the historical period (1966–2005) into two parts: data for 1986–2005 were used for training and data for 1966–1985 were used for validation. A trend-preserving bias correction, the Inter-Sectoral Impact Model Intercomparison Project (ISI-MIP) approach [1], together with a high-quality gridded meteorological dataset based on ground observations (CN05.1), was applied to bias-correct Tmax, Tmin, Pr and Wind by grid point for the training period (1986–2005). According to Hempel et al. [1], the bias-correction was conducted in a two-step approach. The first step corrected the monthly mean data, based on which the second step corrected daily variability.

1) Correction for monthly mean

Following the algorithm, a constant offset *C* was used to bias-correct temperature, which was the difference of 20-year monthly mean temperature of CN05.1 and GCMs:

$$C=\left(\sum_{i=1}^{m=20}T_{i}^{CN05.1}-\sum_{i=1}^{m=20}T_{i}^{GCM}\right)/20,$$

in which *i* represented year, $T_{i}^{CN05.1}$ and $T_{i}^{GCM}$ were monthly mean temperature of CN05.1 and GCMs. In this part, temperature represented Tmax or Tmin.

Multiplicative correction factor *c* was used to bias-correct precipitation and wind, which was the ratio of 20-year monthly mean precipitation/wind of CN05.1 and GCMs. Take precipitation as an example,

$$c=\sum_{i=1}^{m=20}P_{i}^{CN05.1}/\sum_{i=1}^{m=20}P_{i}^{GCM},$$

in which $P_{i}^{CN05.1}$ and $P_{i}^{GCM}$ were the monthly mean precipitation of CN05.1 and GCMs.

2) Correction for daily variability

In the second step, daily variability was corrected by mapping daily residuals. For temperature,

$$\Delta T_{ij}^{GCM}=T_{ij}^{GCM}-T_{i}^{GCM}.$$

The residual temperature $\Delta T_{ij}^{GCM}\;$of year *i*, day *j* was simply the difference between the daily value and its corresponding monthly value.

For precipitation and wind, the daily residual was the ratio between daily value and corresponding monthly value. Take precipitation as an example,

$$\delta P_{ij}^{GCM}=P_{ij}^{GCM}/P_{i}^{GCM}.$$

The variability of daily residuals of the GCMs was adjusted by using some transfer functions. For temperature, we had the function

$$f\left(\Delta T^{GCM}\right)=B\cdot \Delta T^{GCM},$$

where B was the slope of linear regression on the rank ordered daily residuals of CN05.1 data (Δ*T*^*CN*05.1^) and GCMs data (Δ*T^GCM^*).

The daily variability of precipitation and wind was adjusted using nonlinear regression,

$$g\left(\delta {\hat{P}}^{GCM}\right)=\left[a+b\cdot \left\{\delta {\hat{P}}^{GCM}-\delta {\hat{P}}_{min}^{GCM}\right\}\right]\times \left[1-exp\left\{-\frac{\delta {\hat{P}}^{GCM}-\delta {\hat{P}}_{min}^{GCM}}{\tau }\right\}\right],$$

where $\delta {\hat{P}}^{GCM}$ was the rank ordered daily residual data of GCMs, $\delta {\hat{P}}_{min}^{GCM}$ is the lowest value, and a, b and *τ* were the parameters of the function. Superscript ^ represented the data of which the frequency of dry days was corrected (see Ref [1]).

Finally, the bias-corrected data of temperature was:

$${\tilde{T}}_{ij}^{GCM}=C+T_{i}^{GCM}+\Delta {\tilde{T}}_{ij}^{GCM},$$

and precipitation was:

$${\tilde{P}}_{ij}^{GCM}=c\cdot {\hat{P}}_{i}^{GCM}\cdot \delta {\tilde{P}}_{ij}^{GCM},$$

where ${\tilde{T}}_{ij}^{GCM}$ and ${\tilde{P}}_{ij}^{GCM}$ were the bias-corrected data, $\Delta {\tilde{T}}_{ij}^{GCM}$ and $\delta {\tilde{P}}_{\mathrm{ij}}^{\mathrm{GCM}}$ were the residual data after adjusted by mapping functions *f*(Δ*T^GCM^*) and $g(\delta {\hat{P}}^{GCM})$. The correction method of wind speed was the same as that of precipitation.

After that, the correction parameters for the training period were then derived. They were validated for the period 1966–1985. Validation results indicated that parameters based on the training dataset worked fairly well for the validation dataset, and therefore suggested plausible applicability to the future period. These parameters were then applied to bias-correct the future period (2006–2095/2099/2100). More details of the bias correction approach can be found in Ref [1] and more details of the performance of our bias correction can be found in Ref [5].

## Declaration of Competing Interest

The authors declare that they have no known competing financial interests or personal relationships which have, or could be perceived to have, influenced the work reported in this article.

## Appendix Supplementary materials

Image, application 1

Image, application 2
